# Book Reviews

**Published:** 1899-04

**Authors:** 


### BOOK REVIEWS, PAMPHLETS, EXCHANGES.

### BOOK REVIEWS.

[Contributions solicited for review.]

THE PRACTICE OF OBSTETRICS. BY AMERICAN AUTHORS. Edited by Charles Jewett, M.D., Professor of Obstetrics in Long Island College Hospital, Brooklyn, N. Y. In one handsome octavo volume of 763 pages, with 441 engravings in colors and black, and 22 full-page colored plates. Cloth, *net*, $5.00; leather, *net*, $6.00. Lea Brothers & Co., Publishers, Philadelphia and New York.

Several months ago we made mention of the forthcoming publication of this work, and now it is complete before us. It has been produced under the editorial management of Dr. Charles Jewett, of Brooklyn, N. Y., assisted by a number of American collaborators. Most of these latter are men of well-known ability and names familiar to the medical profession, while there are some who, being as yet younger men, have not attained this state of notoriety. As to the book itself, we unhesitatingly affirm that it must commend itself to every reader and student of the medical profession.

Part I. deals with the Anatomy of the Pelvic Organs and the Mammary Glands. In this chapter may be found a thorough anatomical dissertation on these parts. The author has brought into requisition, as can also be said for the remaining portions of the book, some beautiful chromo-lithographs, which always add much to anatomical treatises. Those giving the veins and arteries in the pelvis are works of art. We cannot say that we admire the manner of picturing the mammary glands of a virgin, and a woman who has been pregnant, by giving the photograph of the women's faces, for the same thing could be accomplished by leaving the head absent, or at least by using a mask over the face.

Part II. deals with the Physiology of Pregnancy. In this portion there appear some beautiful plates after His, the well-known anatomist, who has done such excellent work in anatomical embryology. There is also a tabulated "Signs of Pregnancy" representing each month of pregnancy, which commends itself to us.

Part III. Treats of Physiology of Labor.

Part IV. Physiology of the Puerperium.

Part V. Pathology of Pregnancy.

Part VI. Pathology of Labor.

Part VII. Pathology of the Puerperium.

Part VIII. Obstetric Surgery.

But it would be impossible for us to point out all the excellencies which this book certainly possesses. The illustrations throughout are splendid, and if we have any criticism to make it is on the fact that the chapter on Cæsarean Section and Porro's Operation is not illustrated enough in keeping with the remaining portion of the book. Dr. Jewett is certainly to be congratulated, as also the publishers, Messrs. Lea Brothers, for the excellency of the general make-up of the book.

---

The American Year-Book of Medicine and Surgery. Being a Yearly Digest of Scientific Progress and Authoritative Opinion in all Branches of Medicine and Surgery, drawn from Journals, Monographs and Text-Books of the Leading American and Foreign Authors and Investigators. Under the general editorial charge of Geo. M. Gould, M.D. Published by W. B. Saunders, Philadelphia. Price, Cloth, $6.50; Half Mor., $7.50. By Subscription.

This is the Year-Book for 1899, meaning, of course, the compilation of all matter occurring during the year 1898. Like its predecessor, it is a work of great value to every physician in any department of medicine, specialist or not. The title of the book tells just what it contains. It is a volume of 1,032 pages, elegantly bound, and gives within its pages a résumé of all advances made in every department of medicine. Now the physician can readily see of how much value such a work would be to him if he wished to write upon or to study any particular subject. He has before him in a concentrated manner all of importance which has occurred in the domain of medicine during the previous year. One may gather from the contents what the work is expected to contain.

1. General Medicine.
2. General Surgery.
3. Obstetrics.
4. Gynecology.
5. Pediatrics.
6. Pathology.
7. Nervous and Mental Diseases.
8. Orthopedic Surgery.
9. Ophthalmology.
10. Otology.
11. Diseases of Nose and Larynx.
12. Materia Medica and Experimental Therapeutics.
13. Anatomy.
14. Physiology.
15. Legal Medicine.
16. Public Hygiene and Preventive Medicine.
17. Physiologic Chemistry.

The collaborators have done their work well. Throughout the book illustrations are abundant, and most of them works of art. This book should be in the library of every physician.

---

LECTURES ON APPENDICITIS AND NOTES ON OTHER SUBJECTS. By Robert T. Morris, A.M., M.D., Adjunct Professor of Surgery in New York Post-Graduate Medical School and Hospital. Published by G. P. Putnam's Sons, New York.

This little work by Dr. Morris has now been issued for the third time, with, of course, several additions. Most of what occurs in the book had been delivered previously as lectures in the Post-Graduate Medical School, while other portions have appeared at intervals in the various medical journals. It is certainly an excellent little work, and gives the present status of appendicitis operation in a clear and forcible manner. It is of course written by a surgeon, and therefore from the surgical standpoint, and as such is a clear exposition. The general practitioner will of course find many things in it at variance to his own ideas, but we venture to assert that there are more varied opinions on the one subject appendicitis than on any other medical topic. The illustrations representing the pathological and anatomical specimens of the appendix and its contiguous structures, and also the various technique of the operation, have been beautifully executed, and one of distinct value to the book. The author adopts the McBurney blunt dissection operation for entering the abdominal cavity, yet he declares "that in the art of surgery every surgeon is a law unto himself, and that he knows the factors of his own success." This statement will certainly be accepted as absolutely true. Besides the chapters on appendicitis, the author has given some of his original ideas in regard to a number of other subjects, medical and surgical, all of which add much to the value of the book. The publishers have done their work well.

---

AN AMERICAN TEXT-BOOK OF DISEASES OF THE EYE, EAR, NOSE AND THROAT. Edited by G. E. de Schweinitz, A.M., M.D., and B. Alex. Randall, M.A., M.D., Ph.D., of Philadelphia. Published by W. B. Saunders, Philadelphia. Price $7.00 Cloth; $8.00 Sheep.

This is one of the series of American Text-Books recently gotten out by the above well-known publishing house. Like its predecessors, which have been reviewed in these pages, it has much to commend itself. It treats of all of those organs which are usually embraced under one specialty, and is therefore one volume in compact form. This in itself is an advantage. The work was under the editorial management of two men well qualified for the work, one of whom has already given to the profession an excellent Text-Book himself on the eye. The work on the whole is to be commended, yet if there is one criticism we should make it is that the editors have endeavored to make the work *too American*, if such a thing is possible. We mean by this that there are 60 contributors, although in some instances the writer is given but few words to say. For instance, we could not see the propriety of giving to six separate men the treatment of surgical operations on the eyeball and its appendages. The collaborators represent every section of the country, and this was perhaps the intent of the editors. A good number of names we have never seen in print before, but the character of their work shows them well qualified for the undertaking. Diseases of the Conjunctiva, by Dr. Weeks, of New York, and Diseases of the Retina, by Dr. Howe, of Buffalo, should be specially mentioned. The Nose, Throat and Ear Diseases are especially well treated. The chapter on "Diseases of the Accessory Sinuses of the Nose," by Dr. Myles, of New York, and the chapters by Dr. Roe, of Rochester, are especially good. The publishers are to be congratulated for the excellency of their work, and such a Text-Book should readily find favor.

---

FEVER-NURSING. Designed for the Use of Professional and Other Nurses, and especially as a Text-Book for Nurses in Training. By J. C. Wilson, A.M., M.D., of Philadelphia. Published by J. B. Lippincott Company, Philadelphia. Price $1.00.

This is an exceedingly valuable little book. It is the third edition, and its author states in the preface of the first edition the real intent of the work. He says: "The following pages embody the substance of a course of lectures on Fever-Nursing, originally delivered before the Nurse Class at the Philadelphia Hospital. I have sought to treat the subject in plain words, and from the standpoint of the physician; to treat not only *how* fever patients are to be cared for, but also *why* they must be cared for in particular ways." Not only is this book valuable to nurses, but it is also valuable to physicians, especially to those whose clinical work is limited, and who are not connected with some large hospital. The author is exceedingly practical, and above all, clear in his statements. Fever charts are represented wherever necessary, and their manner of use is well shown. Dr. Wilson has certainly done a great service for nurses in writing this little book. We commend it most heartily.

DISEASES OF THE EYE. A Handbook of Ophthalmic Practice, for Students and Practitioners. By G. E. de Schweinitz, A.M., M.D., Philadelphia. Third Edition. Published by W. B. Saunders, Philadelphia. Price, Cloth, $4.00 ; Sheep, $5.00.

This is the third edition of Dr. de Schweinitz's most excellent work on Ophthalmology, and this revision, necessitated within the space of six years, well attests the popularity of the book. This new edition has been thoroughly revised, and much new matter has been added, which enhances even more the value of the work. The original chapters on Physiological Optics, written by Dr. Wallace, have been revised by Dr. Edward Jackson, whose work in this particular branch of ophthalmology has stamped him as an authority. Quite a good deal of space has been given to the discussion of the various micro-organisms which are sometimes found in the eye, a feature which is now necessary for any scientific work on ophthalmology. A goodly number of new illustrations appear in this volume, besides many excellent chromo-lithographic plates. These are always valuable, and add much to any book.

---

THE LIGHT OF REASON. A SOLUTION OF THE ECONOMIC RIDDLE. By A. B. Franklin. Published by Charles H. Kerr & Co., 56 Fifth Ave., Chicago.

This little book of 192 pages treats of the economic principle in the very novel manner of a story in fiction. The questions represented are thus put in an entertaining way, which perhaps makes it better understood. It is an interesting little brochure.

